# From Womb to Tomb: A Life-Course Perspective on the Origins of Non-communicable Diseases

**DOI:** 10.7759/cureus.93168

**Published:** 2025-09-25

**Authors:** Apratim Dev, Ruchika Patra, Shruti Ajay Kale, Leander Pradeep James Marianadin, Sruti Soumya Mohanty, Joel Benny

**Affiliations:** 1 Department of Community Medicine and Family Medicine, All India Institute of Medical Sciences, Bhubaneswar, IND; 2 Department of Burns and Plastic Surgery, All India Institute of Medical Sciences, Bhubaneswar, IND; 3 Department of Medicine and Surgery, Kasbekar Metgud Multispeciality Hospital, Belgaum, IND; 4 Department of Critical Care, Christian Medical College Vellore, Vellore, IND; 5 Department of Physiology, Maharaja Krishna Chandra Gajapati Medical College &amp; Hospital, Berhampur, IND; 6 Department of General Medicine, All India Institute of Medical Sciences, Bhubaneswar, IND

**Keywords:** developmental origins of health and disease, infant, life change events, low birth weight, non-communicable diseases, prenatal exposure delayed effects, social determinants of health

## Abstract

Chronic illnesses such as diabetes, cardiovascular diseases, and respiratory disorders are leading causes of death globally. Although often considered adult-onset, growing evidence links their origins to early developmental exposures, including prenatal, neonatal, and early childhood stages. This review adopts a life-course perspective to examine how factors such as maternal undernutrition, gestational diabetes, low birth weight, early antibiotic use, suboptimal infant feeding, adverse childhood experiences, and childhood obesity influence long-term disease risk through mechanisms like epigenetic modifications, immune and hormonal programming, and metabolic changes. The window from conception through the first two years is especially critical for physiological programming. Evidence from cohort studies supports these associations, though findings are primarily drawn from high-income countries, limiting generalizability to more vulnerable settings. Despite awareness of early-life influence, most prevention remains adult-focused. There is a need for intergenerational strategies spanning preconception to early childhood, alongside school-based interventions and population-level policies. Mobile health technologies and personalized risk profiling offer scalable, cost-effective approaches to early intervention, particularly in underserved communities. The "womb-to-tomb" model emphasizes the urgency of investing in early health to achieve sustainable, equitable prevention of non-communicable diseases (NCDs) throughout life.

## Introduction and background

The top killers of humans across the world include non-communicable diseases (NCDs) like cardiovascular diseases, diabetes mellitus, chronic respiratory diseases, and cancers, which cause about 74% of total deaths in the world [1]. Over 80% of these happen in low- and middle-income countries (LMICs), where already weak health systems are struggling to address the twin burden of infectious diseases and chronic illnesses [2]. NCDs caused more than 41 million deaths in 2021, of which 17 million were premature deaths under the age of 70 years [3]. In addition to mortality, they are the leading cause of disability-adjusted life years (DALYs) and cause a substantial economic burden, especially in LMICs, where disease often leads to household poverty [4].

The burden is not uniform across regions. In high-income countries, cardiovascular diseases prevail as a cause of death, but in LMICs, there is a mixed burden of cardiovascular, metabolic, and respiratory diseases, frequently augmented by malnutrition and infectious diseases [5]. As an example, the International Diabetes Federation estimates that the number of adults affected by diabetes will increase in the future, reaching 783 million people by 2045 compared to 537 million in 2021, with the most significant rise in South Asia and sub-Saharan Africa [6]. In the same way, childhood obesity, which was initially an issue in high-income environments, is currently growing at the fastest rate in LMICs, as the environment has shifted towards diets, physical activity, and urban lifestyles [7]. Such changes highlight the ineffectiveness of the one-sided attention to adult risk factors and also the necessity to examine the determinants that operate much earlier in the life course.

Historically, NCDs were viewed as adult diseases, mostly behaviorally related, with tobacco use, poor diets, alcohol, and physical inactivity being the primary examples. Although such exposures are significant, there is accumulating evidence indicating that the causes of most NCDs date back to fetal life and early childhood [8]. The life-course epidemiology is a reflection of this view because it studies the influence of biological, nutritional, and psychosocial exposures on the developmental windows that determine health over a long period [9].

**Figure 1:**
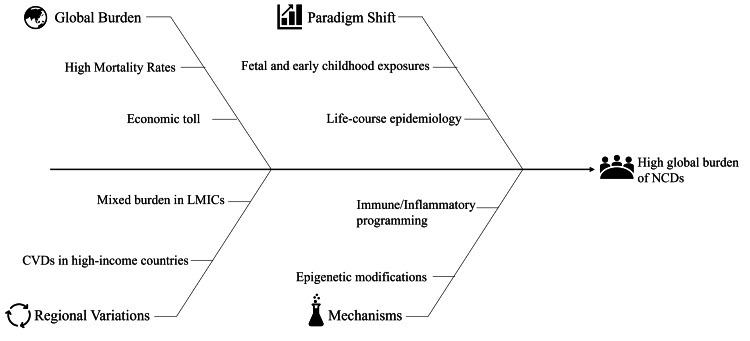
Early-life origins of the global NCD burden Image Credits: Apratim Dev NCD: non-communicable diseases; LMICs: low- and middle-income countries; CVD: cardiovascular disease

One of the most important models in this field is the Developmental Origins of Health and Disease (DOHaD) hypothesis, formulated by David Barker in the 1990s. His initial contributions identified a correlation between low birth weight and increased adult cardiovascular mortality [4], a relationship that was initially disputed but later confirmed by various large-scale longitudinal cohorts. The Avon Longitudinal Study of Parents and Children (ALSPAC, UK) demonstrated links between early-life exposures and cardiometabolic risk [5]; the Pelotas Birth Cohort (Brazil) provided essential insights for a middle-income setting [6]; and the Dutch Hunger Winter Study (Netherlands) offered evidence of a natural experiment of severe prenatal undernutrition [7]. Furthermore, maternal tobacco or alcohol use during pregnancy has been associated with impaired infant neurodevelopment, underscoring how prenatal exposures shape lifelong health trajectories [10]. Evidence from the Brazilian Longitudinal Study of Adult Health also indicates that low birth weight can impair β-cell function and increase insulin resistance in adulthood, reinforcing the metabolic consequences of early growth restriction [11]. Complementing these findings, recent data show that children born small for gestational age often present with adverse lipid profiles, highlighting persistent cardiometabolic risk from infancy through later life [12]. These findings highlight the lasting impact of intrauterine and early postnatal conditions on cardiovascular risk, with lower birth weight associated with higher blood pressure in later life [13]. As shown in Figure 1, NCDs carry a high global burden because of worldwide and regional disparities, early-life exposures, and biological processes that act throughout the life course.

With time, the DOHaD hypothesis has been expanded to include several pathways. DNA methylation and modifications to histones are epigenetic changes that can permanently change gene expression [1]. Early stress is related to the development of cardiometabolic and mental health problems later in life via dysregulation of the hypothalamic-pituitary-adrenal (HPA) axis [14]. The disease susceptibility is also long-term because of immune system programming and low-grade chronic inflammation [3]. Although maternal nutrition and health have been investigated, there is new research evidence indicating the significant paternal and grandparental effects and transgenerational transmission of risk [15]. The results of these studies broadened the area of life-course studies to include maternal effects alone.

Despite these advances, critical research gaps remain. There is disproportionate use of evidence available in high-income countries, and external validity concerns exist in LMICs where risks in early life vary significantly. Molecular mechanisms by which early exposures cause adult diseases are not fully known, restricting the possibility of targeted interventions. Moreover, social factors such as poverty, gender disparities, exposure to the environment, and cultural traditions are not adequately incorporated into the research on DOHaD, although they have a significant effect on health outcomes [16]. These gaps are critical in the formulation of equitable and globally relevant prevention strategies.

Objectives of the review

The review seeks to synthesize the existing body of evidence on the role of early-life exposures, from intrauterine development to childhood, in the development of NCDs throughout the life course. It will then consider the conceptual underpinnings of life-course epidemiology and the DOHaD framework, followed by a critical discussion of some of the major cohort studies and case examples illustrating the connections between early exposures and adult disease outcomes. Also, the review will discuss the biological processes, including epigenetic, metabolic, and endocrine pathways, through which these associations occur. International inequality will also be considered, especially in LMICs, and the issues they experience. Lastly, the review will also assess the prevention strategies throughout the life course, namely, interventions that start at preconception and continue until adolescence. This review highlights the importance of shifting the paradigm of NCD prevention, which is currently based on an adult-centered model, to a life-course, intergenerational model by synthesizing evidence in biological, social, and policy contexts.

Methods

This review narrative was performed to integrate evidence regarding the developmental origins of NCDs. We searched peer-reviewed articles published in English with no date restriction in PubMed, Scopus, and Google Scholar. Our main search terms were combinations of keywords like "developmental origins of health and disease," "life-course epidemiology," "early-life exposure," "prenatal," "neonatal," "childhood," "non-communicable diseases," "cardiovascular," "type 2 diabetes," and "obesity" with relevant Boolean operators. Eligible research involved observational human studies (cohort, case-control, and cross-sectional designs), systematic reviews, and meta-analyses that compared associations of prenatal, neonatal, or childhood exposures with adult NCD outcomes (e.g., cardiovascular disease, type 2 diabetes, obesity, or relevant biomarkers). The studies had to report quantifiable health outcomes to prove relevance and replicability. Experimental animal studies, single case reports, editorials, commentaries, and articles with no original data were excluded. Reference lists of primary papers were searched by hand to find other sources. Evidence was summarized qualitatively, with biological mechanisms, social determinants, and policy implications emphasized in the included studies.

## Review

The concept of life-course epidemiology

Life-course epidemiology is the study of how exposures to biological, behavioral, and psychosocial factors during development interact to cause disease later in life [17]. Instead of viewing adulthood as the major period of NCD development, this framework emphasizes how disorders that start during intrauterine and early childhood life can set the course that continues into adulthood [18]. One of the first hypotheses to do so was the DOHaD. The early evidence linked low birth weight to increased risks of cardiovascular morbidity and mortality later in life [19]. Although this relationship had been contested in the early days, other longitudinal and natural experiment studies later proved that exposures in the prenatal and early childhood periods had direct and enduring impacts on adult health [20].

Several landmark cohorts have advanced this field. A very large longitudinal study in the United Kingdom, which has tracked over 14,000 families since the early 1990s, has associated maternal smoking with reduced offspring lung function and maternal obesity with subsequent adiposity in childhood [21]. A similar birth cohort study in southern Brazil that began in the early 1980s showed close correlations between impaired fetal growth, high adult blood pressure, and the risk of obesity during adolescence [22]. By comparison, the Dutch famine of the winter of 1944-45 offered a natural experiment. Children born during the famine, when exposed to maternal undernutrition during early gestation, were more likely to develop glucose intolerance and cardiovascular disease decades later [23].

Collectively, these studies highlight three conceptual models of life-course epidemiology. The critical period model describes developmental windows (e.g., in utero) where some exposures are long-term in their effects. The accumulation model implies that the risks accumulate over time as a result of repeated bad exposures [24]. A pathway model focuses on how initial disadvantages, including malnutrition, may cause subsequent disadvantages, including lower education or poor adult behavior, which reinforces disease risk [25]. All these models explain the urgency of NCD prevention that should start long before adulthood. Table 1 demonstrates that landmark cohort studies in various contexts are consistent in their findings that early-life exposures are associated with an elevated risk of NCDs in adulthood.

**Table 1:** Landmark cohort studies underpinning the Developmental Origins of Health and Disease (DOHaD) framework ALSPAC: Avon Longitudinal Study of Parents and Children; LMIC: low- and middle-income countries

Study/Setting	Population and Timeline	Key Exposures	Major Findings	Strengths	Limitations	References
UK Longitudinal Cohort (ALSPAC)	>14,000 families, started in 1991	Maternal smoking, maternal obesity	Impaired lung function in offspring; increased childhood adiposity	Large sample, rich genetic & environmental data	Limited generalizability beyond the high-income context	[11]
Pelotas Birth Cohort (Brazil)	>5,000 newborns, started 1982	Low birth weight, rapid infant growth	Higher adult blood pressure and increased adolescent obesity	Long follow-up, valuable LMIC evidence	Attrition and socioeconomic confounding	[2]
Dutch Hunger Winter Study (Netherlands)	Individuals exposed in utero to famine (1944–45)	Maternal undernutrition	Increased risk of glucose intolerance and cardiovascular disease in adulthood	Clear exposure timing, natural experiment	Historical context, limited diversity	[8]

Early-life risk factors

Prenatal Exposures

The intrauterine environment is central to lifelong health. Maternal undernutrition, micronutrient insufficiency, and placental pathology are factors that lead to limited growth and low birth weight, which are always related to increased risks of metabolic syndrome and cardiovascular disease in later adulthood [26]. Long-term follow-up studies in Latin America that compared low-birth-weight and normal-birth-weight individuals indicate that low-birth-weight individuals have impaired insulin sensitivity and are at higher risk of being hypertensive than individuals with normal birth weight [27]. Conversely, maternal obesity and gestational diabetes mellitus (GDM) expose the unborn to surplus glucose and insulin in utero, predisposing them to obesity and type 2 diabetes [28]. Meta-analyses show that children of women with GDM are at considerably increased risk of obesity and glucose intolerance in adolescence, even in the presence of maternal body weight [29]. But a lot of this evidence comes out of high-income nations, and LMIC data are scarce despite the increasing rates of GDM in these contexts. This scarcity partly reflects challenges in LMICs, such as limited access to biomonitoring, inadequate longitudinal surveillance systems, and under-resourced laboratory infrastructure, which constrain high-quality data collection and thereby limit the generalizability of these findings to these settings.

Lifestyle-related prenatal exposures are also influential. Neurodevelopmental impairment and slower motor and cognitive skill acquisition are associated with tobacco use and alcohol intake during pregnancy [30]. These effects are biologically plausible, but interpretation is confounded by factors like poverty and psychosocial stress, which are often confounded with substance use during pregnancy [31]. New evidence points to more prenatal causes of the risk of adult disease. Prenatal exposure to endocrine-disrupting chemicals, including bisphenol A (BPA) and phthalates, has been linked to abnormal hormonal signaling and obesity, diabetes, and reproductive disorders in children [13]. Psychosocial adversity and intimate partner violence-related stress experienced by the mother may affect fetal programming by dysregulating the HPA axis, predisposing the person to subsequent metabolic and mental health problems [21]. Prenatal infections, including those due to influenza or intrauterine infection, can disfavor immune and neurodevelopmental pathways in subsequent children, increasing susceptibility to chronic disorders during adulthood [32]. According to Table 2, prenatal exposures determine the long-term risks of disease due to nutritional, metabolic, and neurodevelopmental pathways.

**Table 2:** Prenatal exposures and their long-term associations with non-communicable diseases ↑: increased, elevated; CVD: cardiovascular disease; LMIC: low- and middle-income countries; GDM: gestational diabetes mellitus

Exposure	Mechanism	Long-term Outcomes	Contextual Notes	References
Maternal undernutrition, micronutrient deficiency, and placental dysfunction	Restricted fetal growth; impaired organ development	↑ Risk of metabolic syndrome, ↑ hypertension, ↑ and CVD	Consistently shown across longitudinal cohorts; strong evidence from Latin America	[15]
Maternal obesity and GDM	Intrauterine hyperglycemia and hyperinsulinemia	↑ Risk of obesity and type 2 diabetes in offspring	Strong evidence from high-income countries; limited LMIC data despite rising prevalence	[6]
Tobacco and alcohol use during pregnancy	Disrupted fetal neurodevelopment; oxidative stress	Impaired motor and cognitive skills; neurodevelopmental delay	Interpretation complicated by confounding (poverty, stress, co-exposures)	[27]

Neonatal and Infant Factors

Premature infant birth and limited fetal development are linked to chronic metabolic changes. Meta-analyses show that preterm-born adults are at higher risk of hypertension, type 2 diabetes, and dyslipidemia [18-20]. These relations indicate that rapid postnatal catch-up growth can lead to poor cardiometabolic phenotypes.

NCD risk has also been associated with the early-life microbiome. Exposure to antibiotics during the first year of life has also been associated with perturbed microbial diversity in infants, which can negatively affect the maturation of the immune system and the regulation of metabolism [33]. Other studies indicate that such children have an increased risk of developing obesity during later childhood, but evidence is inconsistent, and infections that require antibiotics may be the cause of the observed relationships. Feeding practices are particularly influential. Breastfeeding has the benefit of preventing obesity, diabetes, and some cancers in children [2]. The pooled analyses reveal that six months of exclusive breastfeeding decreases the risks of childhood obesity and type 2 diabetes greatly as compared to early formula feeding. In comparison, early formula exposure and high early weight gain during infancy are consistently related to elevated risks of subsequent adiposity and insulin resistance. Notably, the socioeconomic and cultural contexts influence the prevalence rates of breastfeeding, which depicts the interaction of structural and biological processes in the early-life health patterns [34].

Perinatal medical exposures are also being identified as important determinants in addition to nutrition and infection. Babies born by caesarean section have a changed gut microbiota profile compared to those born vaginally, and some researchers have associated the change with increased incidences of obesity, asthma, and allergies in adulthood [11]. Moreover, stressful events such as long stays in neonatal intensive care units (NICUs) and early exposure to procedural stress have been linked to long-term changes in stress regulation and neurodevelopment, implying that medical settings themselves may be a factor in developmental programming [25]. The first 1,000 days, or the time between conception and the first two years of life, is also now regarded as a sensitive period during which metabolic, endocrine, and immune systems are programmed [20]. The health benefits of interventions during this period, such as micronutrient supplementation, infection control, and exclusive breastfeeding promotion, may have long-term health benefits [35]. Table 3 indicates that exposures in early neonatal and infant life affect lifetime NCD risk.

**Table 3:** Neonatal and infant risk factors and their associations with later NCDs NCD: non-communicable disease

Exposure	Mechanism	Long-Term Outcomes	Contextual Notes	References
Preterm birth and restricted fetal growth	Altered organ development; accelerated catch-up growth	Increased risk of hypertension, type 2 diabetes, and dyslipidemia	Supported by meta-analyses; higher risk if combined with rapid postnatal growth	[18]
Early-life antibiotic exposure	Disruption of gut microbiota; impaired immune maturation	Possible increased risk of childhood obesity	Evidence mixed; potential confounding by underlying infections	[21]
Feeding practices (breastfeeding vs. formula feeding)	Metabolic and immune programming; growth regulation	Breastfeeding is protective against obesity, diabetes, and some cancers; formula and rapid infant weight gain increase the risk of later adiposity and insulin resistance	Exclusive breastfeeding is shaped by socioeconomic and cultural factors	[33]
Critical window: first 1,000 days	Integrated metabolic, immune, and endocrine programming	Long-term reduction in NCD risk when interventions are applied	Micronutrient supplementation, infection control, and exclusive breastfeeding yield lifelong benefits	[10]

Childhood Influences

Childhood dietary patterns strongly predict long-term outcomes. Being overweight at an early age (primary school years) is one of the best predictors of the onset of obesity and chronic illnesses in adulthood [24]. The data indicate that the odds of being overweight as a child and developing obesity as an adult are several times higher, regardless of the parental body size, in prospective cohort studies. More long-lasting cardiometabolic health outcomes have been demonstrated in preventive programs that encourage physical activity, proper nutrition, and less screen time in childhood [5]. Infectious exposures during childhood also contribute to NCD pathways. Recurrent diarrhea in childhood has been linked to poor growth, poor cognitive performance, and predisposition to metabolic disease later in life. Moreover, untreated streptococcal infections are still an important cause of rheumatic heart disease in most LMICs, and this demonstrates the persistence of the effect of infections as a precondition to chronic diseases [36].

Lastly, psychosocial stress and adverse childhood experiences (ACEs), including abuse, neglect, and poverty, have a long-term biological and behavioral impact. The literature is consistent that cumulative ACEs are associated with risks of depression, substance use, early death, and cardiovascular disease in adulthood [15]. These effects are believed to be mediated by chronic HPA axis activation and high allostatic load. The interplay between ACEs and social disadvantage leads to overlapping pathways of vulnerability as well [10]. In addition to these known exposures, other childhood determinants are emerging. Inadequate neurodevelopment, worse educational outcomes, and higher adult risks of chronic disease are linked to low levels of cognitive stimulation and unresponsive caregiving in early and middle childhood [37]. Similarly, high screen exposure and low sleep duration in schoolchildren have been associated with obesity, insulin resistance, and worse cardiometabolic parameters. These new risk factors emphasize the importance of the social and behavioral environment, in general, as a determinant of lifelong health [19]. Table 4 shows that exposures during prenatal, neonatal, and childhood have an aggregate impact on long-term predisposition to various NCDs.

**Table 4:** Early-life risk factors and associated adult NCD outcomes GDM: gestational diabetes mellitus; C-section: cesarean section; NICU: neonatal intensive care unit; ACEs: adverse childhood experiences; COPD: chronic obstructive pulmonary disease; NCD: non-communicable disease

Early-Life Stage	Key Risk Factors	Associated Adult NCD Outcomes	References
Prenatal	Maternal undernutrition, GDM, obesity, smoking, alcohol, and environmental toxins	Metabolic syndrome, type 2 diabetes, cardiovascular disease, and neurodevelopmental disorders	[34]
Neonatal and infant	Preterm birth, low birth weight, C-section, NICU stay, lack of breastfeeding, and antibiotics	Insulin resistance, dyslipidemia, obesity, asthma, allergies, impaired immunity	[23]
Childhood	Poor diet, physical inactivity, infections, ACEs, high screen time, inadequate sleep	Type 2 diabetes, cardiovascular disease, COPD, mental health disorders, and cognitive deficits	[14]

Critical synthesis

All the above evidence shows that prenatal insults, neonatal exposures, and childhood adversities all contribute to the developmental programming of NCDs. The power of the life-course paradigm is demonstrated by robust associations, including the protective effect of breastfeeding or the association between exposure to prenatal famine and subsequent diabetes. Simultaneously, less clear results, including the mixed association between exposure to antibiotics and obesity, show how confounding makes interpretation difficult and heterogeneity across settings makes it complex. Instead of one pathway, what is revealed is an interconnected web of influences. Underlying biological pathways that are common to nutritional deprivation, maternal metabolic disorders, early-life infections, and psychosocial stressors and that modify developmental pathways include epigenetic reprogramming, programming of the immune system, and endocrine dysregulation. The recent evidence also points to mitochondrial dysfunction and the gut-brain axis as other mechanisms by which early exposures influence metabolic and cognitive outcomes, reinforcing the multidimensionality of developmental programming.

Yet major limitations persist. The majority of the large-scale cohort data have been generated in high-income countries, which casts doubt on their generalizability to LMICs, where exposures, comorbidities, and social determinants vary extensively. These contexts are generally underfollowed, and even where the data are available, it is hard to distinguish between biological effects and socioeconomic confounding. This skew threatens to contribute to an inadequate understanding of NCD etiology and to compromise the global generalizability of prevailing models. Such gaps reflect the necessity of more integrative and globally inclusive ways. A stronger causal inference would be possible with multi-country birth cohorts that were harmonized with measures of biological, environmental, and social exposures. The inclusion of molecular markers, including epigenomic and metabolomic profiling and social and environmental data, would help explain how risks accumulate over the life course. It is also critical to assess how this knowledge can be translated into policy and practice. Although there is a powerful case that points to early-life determinants, the majority of national NCD strategies continue to be adult-oriented, indicating a gap between science and practice.

Overall, the existing evidence base confirms the life-course approach but also demonstrates apparent research, policy, and practice gaps. The next big step is to close the disciplinary gaps between molecular biology and public health, epidemiology, and social sciences to develop interventions that are not only biologically informed but also contextually relevant. It is only through these gaps that prevention strategies can be transformed out of theoretical potential into realities that can be felt around the globe.

Pathways linking early risk to adult NCDs

Multiple biological pathways are used to explain the mechanisms through which early-life exposures cause adult diseases. Epigenetic changes, including DNA methylation and histone alteration, change the gene expression without altering the genetic sequence and can be maintained into adulthood or even into the next generation [38]. Studies on famine-exposed cohorts show that intrauterine underfeeding induces permanent changes in the methylation of genes involved in metabolism and growth [29], but some studies indicate that these marks diminish with age, casting doubt on whether they can be reversed [29]. An alternative explanation is the so-called thrifty phenotype hypothesis: people who grow up in a time of scarcity acquire energy-saving metabolic patterns that are dysfunctional in environments with abundant calories, resulting in insulin resistance and obesity [7]. Stress-related mechanisms also play an important role. Childhood trauma has been linked to long-term increases in cortisol levels due to dysregulation of the HPA axis, which has been linked to metabolic disorders and mental health disorders [28]. The role of low-grade, chronic inflammation caused by frequent infections or malnutrition is being increasingly acknowledged as a common pathway to atherosclerosis as well as type 2 diabetes and other chronic diseases [39].

**Figure 2:**
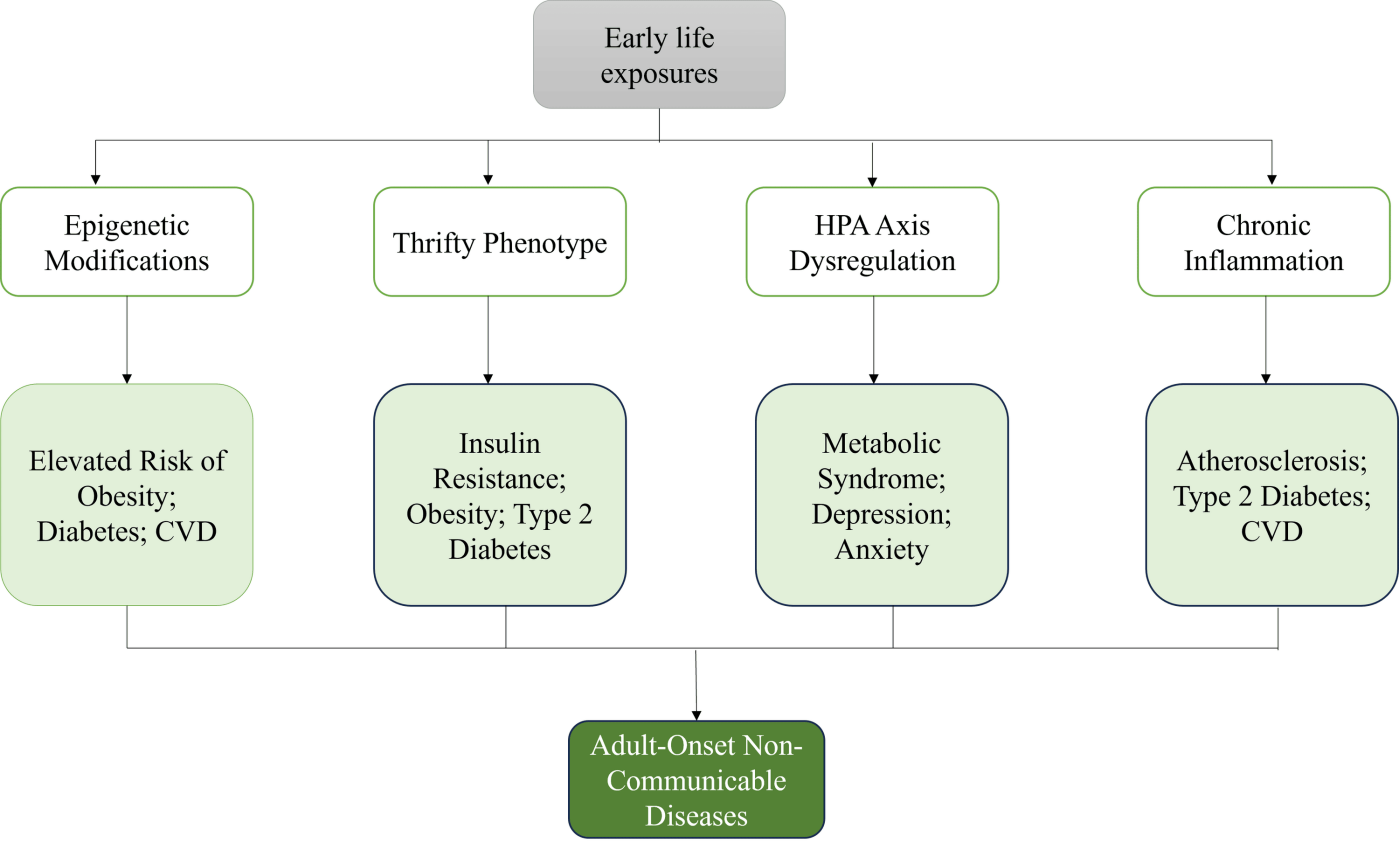
Pathways linking early-life exposures to adult-onset non-communicable diseases Image Credits: Apratim Dev HPA: hypothalamic-pituitary-adrenal; CVD: cardiovascular disease

New data also point to mitochondrial dysfunction as a primary effector of developmental programming. Dysfunctional mitochondrial biogenesis/energy metabolism during development has been associated with elevated risks of obesity, insulin resistance, and neurodegenerative disorders later in life [40]. Simultaneously, changes in the gut-brain axis, which are caused by perturbed microbial colonization, nutrient supply, or early exposure to antibiotics, seem to affect not only metabolic homeostasis but also cognitive outcomes, indicating that it may act in both physical and mental health [16]. Although these mechanisms offer interesting explanations, the relative roles and interplays are not fully understood, reflecting the importance of integrative studies that combine molecular, clinical, and social data. As can be seen in Figure 2, adult NCDs are caused by early-life exposures through epigenetic, metabolic, stress, and inflammatory mechanisms.

Case examples of NCDs

The effects of early exposures can be seen through various NCDs. Cardiovascular risk, comprising changes in cardiac structure and a higher occurrence of ischemic heart disease, has always been associated with low birth weight [30]. However, the relationship is not consistent across populations, and postnatal growth trajectories seem to alter the outcomes, showing the complexity of developmental and environmental interactions, which may partly be due to methodological factors such as differences in study design (e.g., retrospective vs. prospective cohorts), unmeasured or residual confounders like socioeconomic status and maternal nutrition, and variation in the length of follow-up that can affect the detection of late-onset cardiovascular outcomes [24]. In type 2 diabetes, exposure to maternal hyperglycemia in pregnancy and rapid infant weight gain are both risk factors, but the cohort evidence indicates that the effect may depend on geographical location, with South Asians being especially susceptible [41]. Early formula feeding, high infant weight gain, and poor dietary habits are precursors of childhood obesity and metabolic syndrome [42]. Prematurity and household air pollution, especially biomass fuels in low-income contexts, have been linked to respiratory outcomes, including asthma and chronic obstructive pulmonary disease [13].

Finally, the ACEs, including abuse, neglect, and poverty, have also been directly associated with adult mental health disorders and substance use [4]. There is also emerging evidence that early-life adversity is associated with later neurodegenerative disease. As an example, fetal undernutrition and chronic psychosocial stress have been linked to neurodevelopmental impaired trajectories, which can predispose people to dementia and Alzheimer's in old age [43]. Despite the ongoing development of the evidence base, a major gap is the paucity of longitudinal studies that directly link neurodegenerative outcomes to early-life insults over decades, making it apparent that longer follow-up of longer-term studies across a variety of contexts is needed. These case examples show consistent associations but also point to limitations, such as residual confounding and context-to-context variation, which complicate generalization [44].

Global health disparities

NCD burden is especially high in LMICs, where early-life stressors of malnutrition, infections, and suboptimal antenatal care are compounded by rapid urbanization, dietary change, and sedentary living [35]. As an example, South Asia is still experiencing a high prevalence of low birth weight and an increasing prevalence of childhood obesity, leading to a dual burden of malnutrition. Sub-Saharan Africa is a region with ongoing undernutrition and stunting during childhood, coupled with the rising cardiovascular mortality during adulthood. The Latin American cohorts indicate that child survival has not been accompanied by prevention of obesity and diabetes during adolescence [20]. Such inequalities are further complicated by healthcare inequalities such as inadequate access to antenatal services, low immunization coverage, and poor nutrition support. Although there is evidence that early interventions can be effective, national NCD policies in most LMICs are still centered on adult risk modification with limited incorporation of life-course strategies [33]. Other barriers to the adoption of early-life strategies are structural, such as industry interference in the regulation of food and beverages, poor health systems, and competing priorities to address infectious diseases.

Additional global drivers exacerbate these inequities. Climate change has been linked to food insecurity, deficiencies in micronutrients, and exposure to infectious diseases, which enhance early-life vulnerabilities to chronic disease [45]. Health systems are also strained by urbanization and migration, which displace populations to non-traditional diets and expose individuals to obesogenic environments. These structural and environmental pressures, individually and in combination, form a syndemic background in which NCDs overlap with infectious disease burdens, disproportionately impacting the most vulnerable populations of the world [46]. A more holistic policy integration will entail, beyond health sector reforms, intersectoral cooperation in areas of education, gender equity, and poverty reduction.

Prevention strategies

NCDs cannot be addressed effectively by continuing to concentrate on reactive, adult-oriented care, but rather through proactive approaches that encompass the life course [47]. Prevention should start long before conception, as it is known that maternal nutrition, metabolic health, and prevention of adverse exposures determine offspring risk [9]. Nevertheless, structural barriers are often detrimental to these opportunities, such as inadequate access to antenatal care and maternity protection, particularly in LMICs. Studies in South Asia have shown that preconception nutrition interventions can avert poor birth outcomes and the need to target women before pregnancy [40]. Infancy and childhood interventions, including breastfeeding, infection prevention, and vaccination, in addition to their immediate morbidity reduction, prevent later obesity, diabetes, and cognitive impairment [11]. However, the forceful advertisement of breast milk substitutes and inadequate maternity services are among the biggest challenges. The next field of preventive action is schools and communities. There is evidence of cardiometabolic health benefits in interventions to encourage balanced diets, physical activity, and mental health, although sustainability is threatened by obesogenic environments, political opposition, and industry lobbying [24].

Crucially, prevention must be intergenerational. Adolescent empowerment, especially by delaying marriage and increasing education and nutrition, reinforces maternal health and lowers the risk of NCDs in the future generation [7]. The inclusion of DOHaD principles in medical education and primary care guarantees that first-line providers consider these risks at an early age, and digital advancements and models of community empowerment can spread prevention outside of clinical contexts [21]. Finally, the multi-sectoral approach to the collaboration between education, agriculture, and fiscal policies with health priorities is the key to success in the long-term perspective. Nevertheless, even in the case of such strong evidence, policy translation is not even, and part of the reason is that the benefit of early interventions can take decades to become apparent. This short-termism needs to be overcome to establish long-term control of NCDs. Table 5 tells that life-course prevention of NCDs requires stage-specific interventions supported by evidence but limited by structural and policy barriers.

**Table 5:** Life-course strategies for prevention of NCDs GDM: gestational diabetes mellitus; LMICs: low- and middle-income countries; NCDs: non-communicable diseases

Life Stage	Key Interventions	Notes/Challenges	References
Preconception and pregnancy	Maternal nutrition, GDM and hypertension management, avoidance of tobacco/alcohol	Limited antenatal access in LMICs; evidence for preconception nutrition trials	[39]
Infancy and early childhood	Breastfeeding, complementary feeding, vaccination, and infection control	Undermined by weak maternity protection & marketing of breast-milk substitutes	[22]
School age and adolescence	Balanced diet, physical activity, and mental health support	Barriers: obesogenic environments, political resistance, industry lobbying	[44]
Intergenerational and population-level	Adolescent education, delayed marriage, digital health, community empowerment, fiscal policies (taxes, trans-fat bans)	Long timelines discourage policymakers; uneven policy adoption globally	[31]

## Conclusions

The rising burden of NCDs cannot be addressed by targeting adult risk factors alone. A life course approach, beginning in the womb and extending through childhood, adolescence, and adulthood, provides a powerful lens to understand and prevent NCDs. Early-life exposures, whether nutritional, environmental, psychosocial, or epigenetic, can shape lifelong trajectories of health and disease. Recognizing the intergenerational transmission of health risk, interventions must also focus on improving the health of adolescents and young adults, who will become future parents. Building resilient health systems, investing in early education, and ensuring food and environmental security are foundational to a sustainable NCD prevention strategy. Therefore, our efforts for primordial prevention must be directed right at these exposures to crush the seeds of disease before they are planted.

This shift in perspective calls for coordinated action among sectors: linking maternal and child health initiatives with NCD prevention, restructuring health systems to focus on anticipatory care, and addressing the underlying socioeconomic factors that shape health behaviors through generations. The life course approach doesn’t imply only individual prevention-it is about investing in healthier futures for families, communities, and nations. Moving from "womb to tomb" is no longer a metaphor; it is a mandate for global public health.

## References

[1] (2025). WHO: noncommunicable diseases. https://www.who.int/news-room/fact-sheets/detail/noncommunicable-diseases.

[2] Lacagnina S (2020). The Developmental Origins of Health and Disease (DOHaD). Am J Lifestyle Med.

[3] Wagner C, Carmeli C, Jackisch J, Kivimäki M, van der Linden BW, Cullati S, Chiolero A (2024). Life course epidemiology and public health. Lancet Public Health.

[4] Barker DJ (1995). Fetal origins of coronary heart disease. BMJ.

[5] Fraser A, Macdonald-Wallis C, Tilling K (2013). Cohort profile: the Avon Longitudinal Study of Parents and Children: ALSPAC mothers cohort. Int J Epidemiol.

[6] Santos IS, Barros AJ, Matijasevich A, Domingues MR, Barros FC, Victora CG (2011). Cohort profile: the 2004 Pelotas (Brazil) birth cohort study. Int J Epidemiol.

[7] Bleker LS, de Rooij SR, Painter RC, Ravelli AC, Roseboom TJ (2021). Cohort profile: the Dutch famine birth cohort (DFBC)- a prospective birth cohort study in the Netherlands. BMJ Open.

[8] Byrne CD, Phillips DI (2000). Fetal origins of adult disease: epidemiology and mechanisms. J Clin Pathol.

[9] Barouki R, Gluckman PD, Grandjean P, Hanson M, Heindel JJ (2012). Developmental origins of non-communicable disease: implications for research and public health. Environ Health.

[10] Negrão ME, Rocha PR, Saraiva MC (2020). Association between tobacco and/or alcohol consumption during pregnancy and infant development: BRISA Cohort. Braz J Med Biol Res.

[11] Branda JI, de Almeida-Pititto B, Bensenor I, Lotufo PA, Ferreira SR (2022). Low birth weight, β-cell function and insulin resistance in adults: the Brazilian longitudinal study of adult health. Front Endocrinol (Lausanne).

[12] Zamojska J, Niewiadomska-Jarosik K, Kierzkowska B, Gruca M, Wosiak A, Smolewska E (2023). Lipid profile in children born small for gestational age. Nutrients.

[13] Mu M, Wang SF, Sheng J, Zhao Y, Li HZ, Hu CL, Tao FB (2012). Birth weight and subsequent blood pressure: a meta-analysis. Arch Cardiovasc Dis.

[14] Wilkins AT, Reimer RA (2021). Obesity, early life gut microbiota, and antibiotics. Microorganisms.

[15] Masi AC, Stewart CJ (2024). Role of breastfeeding in disease prevention. Microb Biotechnol.

[16] Oddy WH (2012). Infant feeding and obesity risk in the child. Breastfeed Rev.

[17] Zhou Y, Xu Y (2023). Nutrition and metabolism in the first 1000 days of life. Nutrients.

[18] (2025). WHO: obesity and overweight. https://www.who.int/news-room/fact-sheets/detail/obesity-and-overweight.

[19] Tsehay M, Necho M, Mekonnen W (2020). The role of adverse childhood experience on depression symptom, prevalence, and severity among school going adolescents. Depress Res Treat.

[20] Koemel NA, Skilton MR (2022). Epigenetic aging in early life: role of maternal and early childhood nutrition. Curr Nutr Rep.

[21] Alves JG, Alves LV (2024). Early-life nutrition and adult-life outcomes. J Pediatr (Rio J).

[22] Sic A, Cvetkovic K, Manchanda E, Knezevic NN (2024). Neurobiological implications of chronic stress and metabolic dysregulation in inflammatory bowel diseases. Diseases.

[23] Mikulska J, Juszczyk G, Gawrońska-Grzywacz M, Herbet M (2021). HPA axis in the pathomechanism of depression and schizophrenia: new therapeutic strategies based on its participation. Brain Sci.

[24] Pitsavos C, Tampourlou M, Panagiotakos DB, Skoumas Y, Chrysohoou C, Nomikos T, Stefanadis C (2007). Association between low-grade systemic inflammation and type 2 diabetes mellitus among men and women from the ATTICA study. Rev Diabet Stud.

[25] Raisi-Estabragh Z, Cooper J, Bethell MS (2023). Lower birth weight is linked to poorer cardiovascular health in middle-aged population-based adults. Heart.

[26] Ardissino M, Morley AP, Slob EA (2024). Birth weight influences cardiac structure, function, and disease risk: evidence of a causal association. Eur Heart J.

[27] Sheiner E (2020). Gestational diabetes mellitus: long-term consequences for the mother and child grand challenge: how to move on towards secondary prevention?. Front Clin Diabetes Healthc.

[28] Appleton J, Russell CG, Laws R, Fowler C, Campbell K, Denney-Wilson E (2018). Infant formula feeding practices associated with rapid weight gain: a systematic review. Matern Child Nutr.

[29] Pulakka A, Risnes K, Metsälä J (2023). Preterm birth and asthma and COPD in adulthood: a nationwide register study from two Nordic countries. Eur Respir J.

[30] Dai X, Dharmage SC, Lodge CJ (2022). The relationship of early-life household air pollution with childhood asthma and lung function. Eur Respir Rev.

[31] Elmore AL, Crouch E (2020). The association of adverse childhood experiences with anxiety and depression for children and youth, 8 to 17 years of age. Acad Pediatr.

[32] Sahle BW, Reavley NJ, Morgan AJ, Yap MB, Reupert A, Jorm AF (2024). How much do adverse childhood experiences contribute to adolescent anxiety and depression symptoms? Evidence from the longitudinal study of Australian children. BMC Psychiatry.

[33] Morales F, Montserrat-de la Paz S, Leon MJ, Rivero-Pino F (2023). Effects of malnutrition on the immune system and infection and the role of nutritional strategies regarding improvements in children’s health status: a literature review. Nutrients.

[34] Ndubuisi NE (2021). Noncommunicable diseases prevention in low- and middle-income countries: an overview of Health in All Policies (HiAP). Inquiry.

[35] Chadha J, Ahuja P, Mudgil U, Khullar L, Harjai K (2024). Citral and triclosan synergistically silence quorum sensing and potentiate antivirulence response in Pseudomonas aeruginosa. Arch Microbiol.

[36] Hanson MA, Gluckman PD (2014). Early developmental conditioning of later health and disease: physiology or pathophysiology?. Physiol Rev.

[37] Jacob CM, Killeen SL, McAuliffe FM (2020). Prevention of noncommunicable diseases by interventions in the preconception period: a FIGO position paper for action by healthcare practitioners. Int J Gynaecol Obstet.

[38] Victora CG, Bahl R, Barros AJ (2016). Breastfeeding in the 21st century: epidemiology, mechanisms, and lifelong effect. Lancet.

[39] Koletzko B, Brands B, Grote V (2017). Long-term health impact of early nutrition: the power of programming. Ann Nutr Metab.

[40] Itria A, Borges SS, Rinaldi AE, Nucci LB, Enes CC (2021). Taxing sugar-sweetened beverages as a policy to reduce overweight and obesity in countries of different income classifications: a systematic review. Public Health Nutr.

[41] Patton GC, Sawyer SM, Santelli JS (2016). Our future: a Lancet commission on adolescent health and wellbeing. Lancet.

[42] Victora CG, Barros FC (2012). Cohorts in low- and middle-income countries: from still photographs to full-length movies. J Adolesc Health.

[43] Yamada L, Chong S (2017). Epigenetic studies in Developmental Origins of Health and Disease: pitfalls and key considerations for study design and interpretation. J Dev Orig Health Dis.

[44] Soubry A (2015). Epigenetic inheritance and evolution: a paternal perspective on dietary influences. Prog Biophys Mol Biol.

[45] Ouahid H, Sebbani M, Cherkaoui M, Amine M, Adarmouch L (2025). The influence of gender norms on women's sexual and reproductive health outcomes: a systematic review. BMC Womens Health.

[46] Sharma SR, Matheson A, Lambrick D, Faulkner J, Lounsbury DW, Vaidya A, Page R (2022). Individual and community experience of rising burden of non-communicable diseases in two case districts of Nepal: a qualitative exploration. Glob Health Promot.

[47] Sachdeva M, Dugerdil A, Flahault A, Carrara V (2025). Are wearables effective in LMICs?. Public Health Rev.

